# L-Carnitine effect on induced hyperlipidemia on premature rats: fertility profile

**DOI:** 10.25122/jml-2021-0213

**Published:** 2022-01

**Authors:** Khalid Mohammed Karam, Ahmed Saed Alebady, Khalil Gazar Chelab Al-Nailey, Dhia Hussain Jassim Al-Delemi

**Affiliations:** 1. College of Veterinary Medicine, University of Al-Qadisiyah, Al-Qadisiyah, Iraq

**Keywords:** premature rats, cholesterol, hyperlipidemia, L-Carnitine, sperm parameters, lipid profile, CAR-L – Carnitine, CG – control group, CFG1– cholesterol-fed group1, CFG2 – cholesterol-fed group 2, LH – Luteinizing Hormone, FSH – Follicle Stimulating Hormone, hCG – Human chorionic gonadotropin, ROS – reactive oxygen species, NFKB – Nuclear Factor kappa-light-chain-enhancer of activated B cells

## Abstract

This study was designed to investigate the effect of hypercholesterolemia on the reproductive performance of premature male rats and to evaluate the influence of L-Carnitine (CAR) in maintaining their fertility. Sixty rats were divided randomly into three groups. Control group (CG n=20 rats), cholesterol feeding group 1 (CFG1 n=20 rats) fed 1.5% cholesterol with diet for one month, and cholesterol feeding group 2 (CFG2 n=20 rats) fed 1.5% cholesterol with diet + CAR 150 mg/kg body weight (B.W.) given by water for one month. Results showed a significant increase in body weight of CFG1 compared with CG and CFG2. The lipid profile of CFG1 after one month of feeding cholesterol showed a significant increase in serum cholesterol and triglyceride compared with CG and with the group that watered by CAR and CFG2. Results of sperms parameters in CGF2 showed a significant increase in sperms count with sperms live percentage and a significant decrease in sperms abnormalities percentage compared with CGF1 and CG. The hormonal profile showed a significant decrease in serum testosterone levels in rats from CFG1 compared with CFG2 and CG. In conclusion, CAR is a powerful antioxidant that can maintain the parameters of sperms of hypercholesterolemic premature rats, which may enhance the fertilizing ability of subfertile rats that may occur due to hyperlipidemia.

## Introduction

A high-fat diet can affect reproductive efficiency due to the accumulation of free radicals in testicular tissue that causes damages in Sertoli and Leydig cells, which results in functional disorders of the hypothalamic-pituitary-gonadal axis responsible for the clear spermatogenesis process [1, 2]. Using antioxidants as protective agents against the formation of free radicals was known as a strong tool in decreasing oxidative stress and maintaining the biological activities of cells. Using antioxidants has a confirmed positive action on enhancing the fertile properties of semen because they scavenge free radicals that form during the metabolic activities of sperms and leukocytes, decrease the development of premature sperms, and prevent DNA fragmentation of sperms [3].

L-Carnitine (CAR) is a potent antioxidant used as a co-factor for several important mitochondrial enzymes [4]. The therapeutic activity of CAR depends on its action as a scavenger of reactive oxygen species (ROS), metal chelating abilities, capability to repair oxidative damage, and its capacity to regenerate endogenous antioxidants activity [5]. Hyperlipidemia decreases male fertility due to its harmful effect on spermatogenesis [6–9].

The main objective of this study was to evaluate the underlying mechanism of the ameliorative effects of L-carnitine, enhancing the reproductive performance of mature hyperlipidemic premature male rats.

## Material and Methods

The present study was performed on 60 premature albino male rats; their ages were around 4 weeks, with a body weight ranging between 75–100 grams. Rats were obtained from the College of Veterinary Medicine/University of Al-Qadisiyah and were fed and housed under standard nutrition and environment during the whole experimentation days.

Rats were divided randomly into two main groups: control group CG (20 rats) and treatment group (40 rats). The treatment group was subdivided into two groups (20 rats for each). Group 1 (CFG1) cholesterol-fed group were fed on a diet supplemented with 1.5% cholesterol alone while rats from treatment group 2 (CFG2) were fed on a diet supplemented with 1.5% cholesterol (BDH, England) + CAR (AMS, USA) 150 mg/kg body weight given by water, from day 30 to day 60 of age. Rats in the control group were fed on a diet that did not contain cholesterol or CAR from day 30 to day 60 of age. One day after ceasing the cholesterol supplement (on day 61 of age), the weights of rats in all groups were checked, and blood samples were collected to measure serum total cholesterol and triglyceride. The rats were sacrificed, and specimens from the liver and testis of all groups were fixed in 10% formalin for histology [10]. Semen samples were taken from the tail of the epididymis and checked for semen analysis [11].

Tissue specimens, including liver, testes, and epididymis, are extracted by scissors and collected at the end of the study to perform a histological examination of the collected specimens. A sequence of successive histological processing steps was made, as shown in this study [12].

The statistical package for social sciences (SPSS) was used to calculate the statistical analysis. The statistical analysis of physiological and histological parameters was done using Chi-square [13].

## Results

Weights of rats of all groups at day 30 were 92.87±7.21 g, 94.87±6.33 g, and 95.87±5.73 g for CFG1, CFG2, and CG, respectively. After 30 days of cholesterol feeding (at day 61 of age), the weights for groups were 182.4±12.32 g, 147.4±12.32 g, and 133.96±10.43 g for CGF1, CGF2, and CG, respectively. The weight of CGF1 was significantly higher compared with CGF2 and CG, as shown in Table 1.

**Table 1.** Bodyweight (B.W.) of all groups before and after feeding cholesterol (M±SD).

**Groups**	**CFG1**	**CFG2**	**CG**
**B.W at 1 month of age (grams)**	92.87±7.21	94.87±6.33	95.87±5.73
**B.W after 2 months of age (grams)**	182.4±12.32^a^	147.4±12.32^c^	133.96±10.43^c^

*ac – significant difference in rows (P<0.01).

Table 2 represents the measures of the lipid profile of CFG1 and CFG2 after one month of cholesterol feeding (at day 61 of age) and the lipid profile of CG. The serum cholesterol for CFG1 was 126.15±5.91, and serum triglyceride was 176.06±3.56. For CFG2, serum cholesterol was 107.34±4.12, and serum triglyceride was 111.77±6.63. Serum cholesterol for the control group was 95.32±3.85, and serum triglyceride was 98.51±3.76, with significant differences between all groups.

**Table 2.** Lipid profile (cholesterol and triglyceride mg/dl) for all groups (M±SD).

**GROUPS**	**CFG1**	**CFG2**	**CG**
**Cholesterol**	126.15±5.91^a^	107.34±4.12^b^	95.32±3.85^c^
**Triglyceride**	176.06±3.56^a^	111.77±6.63^b^	98.51±3.76^c^

*ab – significant difference in rows (P<0.05); *bc – significant difference in rows (P<0.05); *ac – significant difference in rows (P<0.01).

Sperm parameters after 30 days of cholesterol feeding: sperms count in 1ml of semen were 99.55±7.62×10^6^, 121.05±8.25×10^6^, and 115.27±4.02×10^6^ for CFG1, CFG2, and CG, respectively, with significant differences between groups. Live sperm percentages for CFG1, CFG2, and CG were 74.32±4.38, 80.25±4.51, and 81.13±5.31 percent, respectively, with a significant difference between CGF1 compared with CFG2 and CG. Abnormal sperms percentages were 6.25±1.32, 4.73±1.37, and 3.37±1.92 percent for CFG1, CFG2, and CG, respectively, with significant differences between groups, as shown in Table 3.

**Table 3.** Sperm parameters of all groups at day 61 of age.

**Groups**	**Sperm count ×10^6^**	**Live %**	**Abnormal sperms %**
**CFG1**	99.55±7.62^c^	74.32±4.38^a^	6.25±1.32^a^
**CFG2**	121.05±8.25^a^	80.25±4.51^b^	4.73±1.37^b^
**CG**	115.27±4.02^b^	81.13±5.31^b^	3.37±1.92^c^

*ab – significant difference in columns (P<0.05); *bc – significant difference in columns (P<0.05); *ac – significant difference in columns (P<0.01).

The hormonal profile of all groups was recorded at day 61 of age. Serum LH levels were 15.31±1.20, 16.95±1.18, and 6.34±1.17 mIU/ml in CFG1, CFG2, and CG, respectively, with a significant difference between CFG1 and CFG2, compared with CG. Serum FSH levels were 11.77±1.82, 13.29±2.78, and 8.23±2.77 mIU/ml for CFG1, CFG2, and CG, respectively, with a significant difference between CFG1 and CFG2, compared with CG. Serum testosterone levels were 4.24±0.42, 5.66±0.53, and 7.81±1.89 ng/ml for CFG1, CFG2, and CG, respectively, with a significant difference between CGF1 and CFG2, and CG as in Table 4.

**Table 4.** Hormonal profile of all groups at day 61 of age (M±SD)

**Parameters**			
**Groups**	**LH (mIU/ml)**	**FSH (mIU/ml)**	**Testosterone (ng/ml)**
**CFG1**	15.31±1.20^a^	11.77±1.82^a^	4.24±0.42^c^
**CFG2**	16.95±1.18^a^	13.29±2.78^a^	5.66±0.53^b^
**CG**	6.34±1.17^c^	8.23±2.77^c^	7.81±1.89^a^

*ab – significant difference in columns (P<0.05); *bc – significant difference in columns (P<0.05); *ac – significant difference in columns (P<0.01).

**Figure 1.**
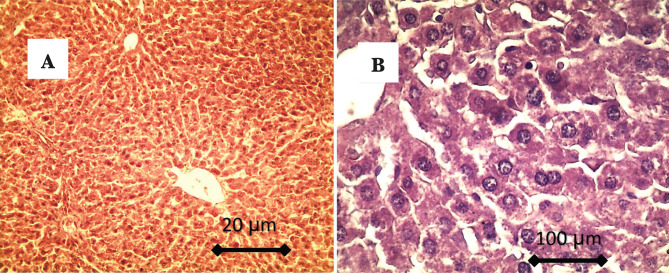
A – showed normal hepatic tissue, characterized by the presence of radially arrangement of hepatocytes around the normal central vein; B – In higher magnification, the hepatocytes showed hexagonal and normal shape.

Histological findings of the liver in CG showed normal hepatic tissue, characterized by radially arranged hepatocytes around the normal central vein. The hepatocytes showed hexagonal and normal shape in higher magnification, as shown in Figure 1 AB.

**Figure 2.**
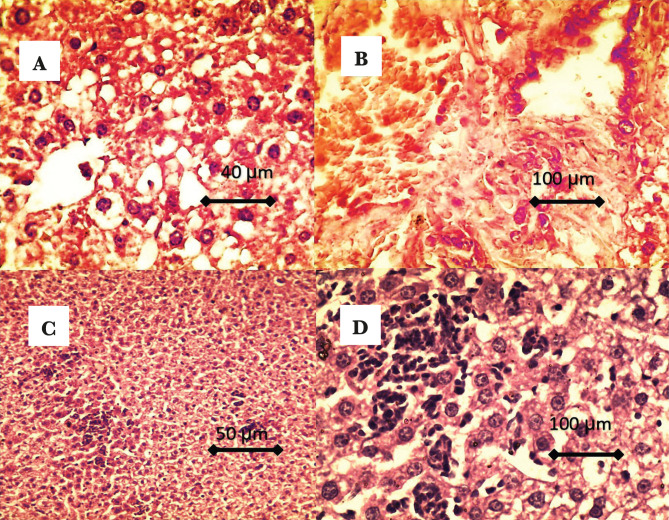
A – Showed loss of hepatic architecture, marked vacuolation of the hepatocyte, presence of fatty change within hepatocyte (hepatocyte shown as signet-like shape); B – The bile duct showed congestion with hyperplasia; C and D – There is infiltration (aggregation) of the inflammatory cells (mainly macrophage) within hepatic tissue, and the hepatocytes showed as binucleated.

After one month of cholesterol administration, histological findings of CFG1 livers showed congestion of the central vein and loss of hepatic architecture; the bile duct showed congestion with hyperplasia (Figure 2 AB). Marked vacuolation of the hepatocyte, presence of fatty change within hepatocyte, hepatocyte showed as binucleated. Also, there is infiltration (aggregation) of the inflammatory cells (mainly macrophage) within hepatic tissue, hepatocyte shown as a signet (Figure 2 CD).

**Figure 3.**
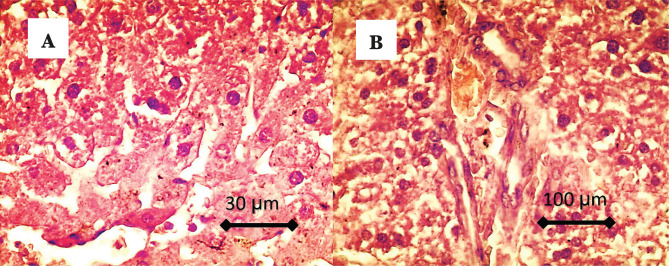
A – The histological findings of the liver showed the presence of hepatic architecture (radial arrangement of hepatocyte around normal central vein), presence of dilation of the sinusoid, mild proliferation of the Kupffer cells, and the hepatocyte showed normal hexagonal shape; B – There is congestion and mild hyperplasia of the bile duct.

While in the CFG2 (CAR) group, histological findings of the liver show the presence of hepatic architecture (radial arrangement of hepatocyte around normal central vein), presence of dilation of the sinusoid, mild proliferation of the Kupffer cells, the hepatocyte showed normal hexagonal shape, congestion and mild hyperplasia of the bile duct (Figure 3 AB).

**Figure 4.**
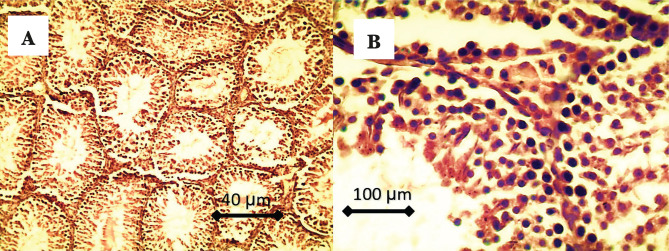
A – There is complete spermatogenesis, and the seminiferous tubules showed compact, circular, and normal shapes; B – Higher number of spermatogonia, primary and secondary spermatocytes, and spermatids can be seen in the lumen of seminiferous tubules. Also, there are high numbers of Leydig cells in the interstitial tissue.

**Figure 5.**
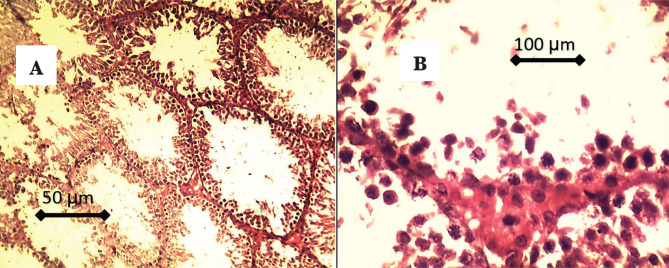
A – There is suppression of spermatogenesis characterized by the absence of sperms in the lumen of the seminiferous tubules, which was very wide; B – Vacuolation of spermatogonia, few numbers of primary and secondary spermatocytes, few numbers, and vacuolation of Leydig cells in the interstitial tissue.

Histological findings of testicular tissue in CG are characterized by complete spermatogenesis, and the seminiferous tubules showed compact, circular, and normal shapes. A higher number of spermatogonia, primary and secondary spermatocyte, and spermatids can be seen in the lumen of seminiferous tubules, as represented in Figure 4 AB,

Histological findings of testes in cholesterol feeding group (CFG1) showed suppression of spermatogenesis characterized by vacuolation of spermatogonia, with few numbers of primary and secondary spermatocytes, absence of sperms in the lumen of the seminiferous tubules which showed very wide, few numbers of Leydig cells in the interstitial tissue with presence of adipose tissue, as shown in Figure 5 AB.

**Figure 6.**
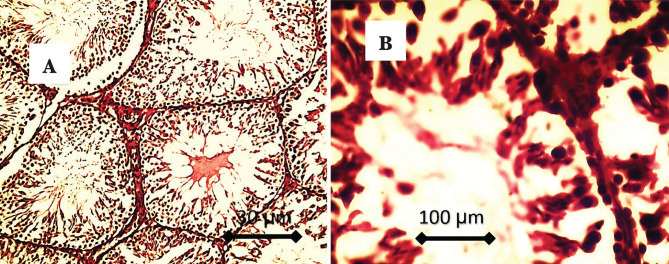
A – There is complete spermatogenesis characterized by the presence of spermatogonia, high numbers of spermatocytes, spermatids, and spermatozoa in the lumen of seminiferous tubules; B – Higher magnification showed mild vacuolation of spermatogonia in a few seminiferous tubules and proliferation of Leydig cells in the interstitial tissue.

In CFG2, there is complete spermatogenesis characterized by the presence of spermatogonia, high numbers of spermatocytes, and spermatids in the lumen of seminiferous tubules. There is mild vacuolation of spermatogonia in a few seminiferous tubules and proliferation of Leydig cells in the interstitial tissue, as seen in Figure 6 AB.

## Discussion

Body weights of CFG1 rats increased significantly compared with CFG2 and CG after one month of cholesterol feeding due to high lipid intake. This causes the accumulation of lipids in the bodies and internal organs of rats, as represented by the increase of body weight, and induces fatty changes of hepatocytes such as vacuolation and loss of architecture. Moreover, blood levels of total cholesterol and triglyceride increased significantly in CFG1 and CFG2 compared with CG after one month of high lipid intake, as shown in Table 2. Those results resemble other studies [14–20] that fed rats and mice with 0.5–1% cholesterol for several weeks and gained body weight and hypercholesterolemia with hypertriglyceridemia.

This study was performed to induce partial infertility by feeding a high lipid content diet that causes hyperlipidemia that affects the spermatogenesis process. Our results meet the goal of reducing rats’ fertility, as shown in Table 3. The results are similar to many previous data obtained from feeding high cholesterol diet to lab animals (mice, rats, rabbits) which caused detrimental effects on testicular tissue and functions such as spermatogenesis, steroidogenesis, epididymal sperm maturation process, sperm quality parameters, sperm fertilizing capacity and fertility index [20–26]. Other research [9, 27, 28] suggested that feeding a high cholesterol diet for several weeks caused an adverse effect on the secretory functions of Leydig and Sertoli cells reducing sperm concentration and motility percentages and increasing abnormal sperm morphology. Ouvrier *et al.* [25] suggested that high cholesterol diet intake can alter the epididymal epithelium structure due to the accumulation of cholesterol droplets in the smooth muscles that lines the epididymal epithelium weakening the peristaltic movement of the epididymis delaying sperms maturation and progression. Table 4 showed that high lipid diet intake decreased serum levels of testosterone. Similar results were recorded in another study [29], which mentioned that hypercholesterolemia inhibited the steroidogenesis process in testis by modulating the bioactive peptides of the renin-angiotensin system that occur in testicles which leads to decreased testosterone production. Tanaka *et al.* [21] mentioned that hypercholesterolemia reduces rats’ serum testosterone levels due to the reduction in testicular LH\HCG binding. Many types of research on humans [30–33] reported a negative correlation between serum triglyceride levels and serum testosterone levels. They mentioned that hypercholesterolemia leads to elevated serum triglyceride levels, which have deleterious effects on spermatogenesis, decrease sperms motility, and decrease serum testosterone levels in infertile men. Our results showed the same data in a rat model. Hypercholesterolemia also causes damage to testicular tissue due to the excessive formation of free radicals that have a cytotoxic effect on spermatozoa [22, 34–36]. Previous studies indicated that a diet supplemented with (antioxidants and/or agents that enhances lipid metabolism) could maintain the reproductive functions of the testis in hypercholesteremic rats [21, 33, 34]. L-Carnitine is known to have antioxidant [37] and anti-inflammatory [38] properties. L-Carnitine can easily cross the biological membranes because of its small size and high lipophilicity [39]. L-Carnitine can improve mitochondrial functions given its ability to stimulate Sirtuin 1 and 3 [40–42], quench free radicals and inhibit ROS generators.

Furthermore, it down-regulates the CAR-dependent pro-inflammatory NF-kB pathway [38]. Our study showed significant testicular histological protection in the hyperlipidemic group of rats treated with CAR compared with the not-treated hyperlipidemic group. This may be attributed to CAR’s ability to scavenge many free radicals such as singlet oxygen, H_2_O_2_, and hydroxyl radicals which formed excessively during lipid peroxidation and metabolic processes [43]. This study showed good histological protection for spermatogenic layers in the CAR treated group compared with the non-CAR treated group, reflecting the better concentration of sperms and the lesser percentage of sperms abnormalities in the treated group.

## Conclusions

According to our study’s results and discussions, oral administration of CAR (150 mg/kg.B.W) with diet established lowering ROS has a considerable favorable influence on male reproductive function by increasing sperm parameters. In addition to being a hepato-protective agent and having an anti-hyperlipidemic effect by lowering blood cholesterol and triglyceride levels, dealing of CAR to premature male rats resulted in the greatest reproductive profile.

## Acknowledgments

### Conflict of interest

The authors declare no conflict of interest.

### Ethical approval

The approval for this study was obtained from the Ethics Committee of the College of Veterinary Medicine, University of Al-Qadisiyah, under Ref. number 543/2018.

### Personal thanks

The authors would like to thank the institute of lab animals at the University of Al-Qadisiyah for their cooperation in achieving this study.

### Authorship

KMK and ASA contributed to conceptualizing, KMK and DHA contributed to the methodology, DHA contributed to writing the original draft, KMK contributed to editing the manuscript, ASA and KGA contributed to data collection, data analysis, and histopathological configurations.
